# Supplementary material for: Raw Milk-Induced Protection against Food Allergic Symptoms in Mice Is Accompanied by Shifts in Microbial Community Structure
Source: Int J Mol Sci. 2021 Mar 26;22(7):3417. doi: 10.3390/ijms22073417 (PMC8037148; doi:10.3390/ijms22073417)
Supplement: Supplementary file 1 [file ijms-22-03417-s001.zip › Supplementary figure legends.docx]

**SUPPLEMENTARY FIGURE LEGENDS**

**Figure S1. Rarefaction curves of alpha diversity metrics.** The observed metrics were (**A**) Shannon diversity and (**B**) number of Operational Taxonomic Units (OTUs). Each colored line represents the microbiome of a single mouse fecal sample. For statistical analyses in the main manuscript, sequence data were rarefied to a depth of 7,000 sequences per sample, a depth at which Shannon diversity was saturated.

**Figure S2.** **Pasteurized milk-treated mice had increased bacterial richness compared to raw milk-treated mice at day -1.** Alpha-diversity indices examined were (**A**) Shannon, (**B**) Simpson’s, (**C**) richness, and (**D**) evenness at the taxonomic level of genus. Measurements were based on 16S rRNA gene amplicon sequence datasets rarefied to 7,000 sequences per sample. One-way ANOVA results and Tukey post-hoc test adjusted *P*-values shown: **P* < 0.05. PBS, phosphate-buffered saline; raw, raw cow’s milk; pasteurized, pasteurized cow’s milk; skimmed, skimmed raw cow’s milk; pasteurized + ALP, pasteurized milk spiked with alkaline phosphatase.

**Figure S3.** **No differences in butyrate-producing genera between treatment groups were observed at day -1.** The relative abundance of (**A**) putative butyrate-producing taxa and (**B**) total butyrate-to-total SCFA ratio examined in the different treatment groups at day -1. No significant differences between groups were observed, as indicated by Kruskal-Wallis tests. PBS, phosphate-buffered saline; raw, raw cow’s milk; pasteurized, pasteurized cow’s milk; skimmed, skimmed raw cow’s milk; pasteurized + ALP, pasteurized milk spiked with alkaline phosphatase; KW, Kruskal-Wallis; SCFA, short-chain fatty acids.

**Figure S4.** **Alpha diversity indices in control and experimental groups at day 31.** Alpha-diversity indices examined were (**A**) Shannon, (**B**) Simpson’s, and (**C**) evenness at the taxonomic level of genus. Measurements were based on 16S rRNA gene amplicon sequence datasets rarefied to 7,000 sequences per sample. PBS, phosphate-buffered saline; OVA, ovalbumin; raw, raw cow’s milk; pasteurized, pasteurized cow’s milk; skimmed, skimmed raw cow’s milk; pasteurized + ALP, pasteurized milk spiked with alkaline phosphatase.

**Figure S5.** **Taxonomic feature selection using a machine learning random forest model (Boruta) at day 31.** Fourteen genus-level taxa of importance for differentiating between control and experimental groups at day 31 were identified. Taxa included *Bifidobacterium*, *Parasutterella*, *Candidatus Stoquefichus*, *Lachnospiraceae* NK4A136 group, *Ruminiclostridium* 5, *Lachnospiraceae* UCG-001, *Lachnospiraceae* UCG-008, *Erysipelotrichaceae* (Other), *Oscillibacter*, *Eubacterium nodatum* group, *Clostridiales vadin* BB60 group, *Blautia*, *Butyricicoccus*, and *Intestinimonas*.
