# Supplementary material for: Raw Milk-Induced Protection against Food Allergic Symptoms in Mice Is Accompanied by Shifts in Microbial Community Structure
Source: Int J Mol Sci. 2021 Mar 26;22(7):3417. doi: 10.3390/ijms22073417 (PMC8037148; doi:10.3390/ijms22073417)
Supplement: Supplementary file 1 [file ijms-22-03417-s001.zip › Supplementary tables.docx]

**SUPPLEMENTARY TABLES**

**Table S1.** Average total count and percent relative abundance of sequences of individual fecal taxa between the different groups at day -1.

| **Taxonomic Levels** | **PBS ^1^**  **Count**  **(% RA)** | **Raw ^2^**  **Count**  **(% RA)** | **Pasteurized ^3^**  **Count**  **(% RA)** | **Skimmed ^4^**  **Count**  **(% RA)** | **Pasteurized + ALP ^5^**  **Count**  **(% RA)** | ***P*-value *** | **FDR  *P-v*alue *** | **Dunn’s**  **post-hoc tests ^#^** |
| --- | --- | --- | --- | --- | --- | --- | --- | --- |
| **Phylum** | | | | | | | | |
| Actinobacteria | 210.42  (0.49) | 190.62 (0.46) | 492.5  (1.09) | 267.12  (0.67) | 185.5  (0.50) | **0.019** | 0.379 | **1 v 3, 2 v 3,**  **3 v 5** |
| Bacteroidetes | 9,216.42 (20.40) | 7,919.87 (18.89) | 11,136.12 (23.61) | 9,662.62  (23.39) | 7,706.37  (19.53) | 0.181 | 0.480 | NS |
| Deferribacteres | 403.85  (1.06) | 565.87 (1.38) | 714.62  (1.79) | 211.87  (0.68) | 426.75  (1.13) | 0.400 | 0.551 | NS |
| Firmicutes | 30,949.35 (76.60) | 32,302.87 (78.34) | 31,167.37 (72.09) | 28,050.87  (74.08) | 27,851.12  (77.57) | *0.079* | 0.379 | NS |
| Proteobacteria | 536.714 (1.24) | 384  (0.90) | 613.37  (1.35) | 452.12  (1.12) | 435.12  (1.19) | **0.043** | 0.379 | **1 v 3, 2 v 3** |
| Tenericutes | 80.28  (0.16) | 1.25  (0.00) | 15.12  (0.03) | 10.5  (0.02) | 14.75  (0.04) | 0.138 | 0.480 | NS |
| **Family** | | | | | | | | |
| Atopobiaceae | 0.86  (0.00) | 4.75  (0.01) | 12.88  (0.03) | 2.50  (0.01) | 10.63  (0.04) | 0.722 | 0.772 | NS |
| Bacteroidaceae | 2,050.29  (4.43) | 1,087.13  (2.59) | 2,396  (5.01) | 1,983.88  (4.73) | 1,350.63  (3.14) | 0.245 | 0.566 | NS |
| Bifidobacteriaceae | 16.29  (0.04) | 0.25  (0.00) | 221.38  (0.49) | 0.38  (0.00) | 0.00  (0.00) | 0.498 | 0.716 | NS |
| Burkholderiaceae | 340.64  (0.76) | 307.88  (0.73) | 467.50  (1.01) | 255.75  (0.61) | 241.13  (0.65) | 0.361 | 0.621 | NS |
| Clostridiaceae 1 | 256.57  (0.57) | 213.88  (0.50) | 299.00  (0.66) | 402.63  (0.93) | 156.75  (0.40) | 0.351 | 0.609 | NS |
| Clostridiales vadin BB60 group | 292.71  (0.72) | 195.00  (0.47) | 684.00  (1.53) | 253.63  (0.62) | 227.13  (0.62) | **0.038** | 0.566 | **2 v 3, 3 v 4,**  **3 v 5** |
| Deferribacteraceae | 403.86  (1.06) | 565.88  (1.38) | 714.63  (1.79) | 211.88  (0.68) | 426.75  (1.13) | 0.347 | 0.605 | NS |
| Desulfovibrionaceae | 163.43  (0.41) | 61.13  (0.14) | 89.00  (0.21) | 185.63  (0.48) | 180.38  (0.50) | *0.065* | 0.566 | NS |
| Eggerthellaceae | 191.71  (0.45) | 184.88  (0.45) | 243.13  (0.53) | 262.88  (0.66) | 174.25  (0.46) | 0.127 | 0.566 | NS |
| Enterobacteriaceae | 27.1  (0.06) | 11.38  (0.03) | 49.13  (0.12) | 4.75  (0.01) | 9.00  (0.02) | *0.054* | 0.566 | NS |
| Erysipelotrichaceae | 257.14  (0.58) | 94.25  (0.22) | 298.38  (0.62) | 204.38  (0.49) | 127.50  (0.35) | **0.040** | 0.566 | **1 v 2, 2 v 3** |
| Lachnospiraceae | 22,931.93  (57.69) | 24,623.50  (60.13) | 21,619.13  (50.60) | 20,269.75  (54.99) | 20,975.88  (58.89) | 0.370 | 0.627 | NS |
| Lactobacillaceae | 4,625.57  (10.67) | 4,643.88  (10.85) | 5,882.63  (13.08) | 4,638.25  (11.10) | 4,187.13  (11.20) | 0.583 | 0.755 | NS |
| Muribaculaceae | 5,443.86  (12.04) | 5,139.38  (12.22) | 6,394.50  (13.45) | 5,521.88  (13.45) | 4,833.88  (12.50) | 0.654 | 0.755 | NS |
| Peptococcaceae | 50.50  (0.12) | 39.50  (0.09) | 54.63  (0.12) | 52.38  (0.14) | 45.38  (0.13) | 0.136 | 0.566 | NS |
| Prevotellaceae | 150.86  (0.33) | 250.38  (0.60) | 552.88  (1.09) | 424.13  (0.98) | 282.13  (0.65) | 0.157 | 0.566 | NS |
| Rikenellaceae | 1,530.79  (3.52) | 1,426.38  (3.45) | 1,744  (3.95) | 1,699.25  (4.15) | 1,217.13  (3.18) | 0.319 | 0.569 | NS |
| Ruminococcaceae | 2,458.57  (6.06) | 2,432.50  (5.93) | 2,239.50  (5.28) | 2,157.75  (5.62) | 2,070.00  (5.82) | 0.326 | 0.574 | NS |
| **Genus** | | | | | | | | |
| *Alistipes* | 1,311.14  (3.04) | 1,276.00  (3.08) | 1,532.50  (3.50) | 1,452.63  (3.55) | 1,069.25  (2.82) | 0.237 | 0.563 | NS |
| *Alloprevotella* | 140.43  (0.30) | 238.63  (0.57) | 530.25  (1.05) | 404.88  (0.93) | 266.88  (0.62) | 0.188 | 0.563 | NS |
| *Anaerotruncus* | 324.86  (0.82) | 443.88  (1.07) | 294.13  (0.70) | 260.25  (0.68) | 318.63  (0.89) | 0.176 | 0.563 | NS |
| *ASF356* | 680.07  (1.73) | 524.25  (1.26) | 444.75  (1.05) | 312.88  (0.86) | 594.00  (1.58) | 0.127 | 0.563 | NS |
| *Bacteroidaceae;* Other | 54.00  (0.12) | 29.88  (0.07) | 60.38  (0.13) | 53.25  (0.13) | 36.75  (0.09) | 0.293 | 0.563 | NS |
| *Bacteroides* | 1,996.29  (4.31) | 1,057.25  (2.52) | 2,335.63  (4.88) | 1,930.63  (4.60) | 1,313.88  (3.06) | 0.323 | 0.564 | NS |
| *Bifidobacterium* | 15.71  (0.04) | 0.13  (0.00) | 213.13  (0.47) | 0.38  (0.00) | 0.00  (0.00) | 0.847 | 0.871 | NS |
| *Bilophila* | 162.14  (0.41) | 60.25  (0.13) | 87.88  (0.21) | 183.75  (0.48) | 178.13  (0.49) | 0.115 | 0.563 | NS |
| *Blautia* | 288.43  (0.71) | 427.63  (1.05) | 331.13  (0.74) | 228.00  (0.62) | 390.63  (0.91) | 0.681 | 0.760 | NS |
| *Butyricicoccus* | 151.93  (0.39) | 148.75  (0.38) | 91.38  (0.22) | 141.50  (0.36) | 126.63  (0.38) | 0.538 | 0.754 | NS |
| *Candidatus Arthromitus* | 248.57  (0.55) | 205.25  (0.48) | 289.13  (0.64) | 387.50  (0.90) | 150.63  (0.38) | 0.482 | 0.708 | NS |
| *Candidatus Stoquefichus* | 66.43  (0.14) | 0.50  (0.00) | 63.5  (0.12) | 39.13  (0.09) | 7.63  (0.02) | *0.096* | 0.563 | NS |
| *Clostridiales vadin* BB60 group; Other | 292.71  (0.72) | 195.00  (0.47) | 684.00  (1.53) | 253.63  (0.62) | 227.13  (0.62) | *0.072* | 0.563 | NS |
| *Coriobacteriaceae* UCG-002 | 0.79  (0.00) | 4.38  (0.01) | 12.63  (0.03) | 2.50  (0.01) | 9.88  (0.03) | 0.672 | 0.760 | NS |
| *Enterorhabdus* | 163.14  (0.38) | 156.25  (0.37) | 209.13  (0.45) | 220.88  (0.56) | 153.75  (0.41) | 0.149 | 0.563 | NS |
| *Erysipelatoclostridium* | 21.43  (0.05) | 28.25  (0.06) | 134.25  (0.28) | 78.25  (0.18) | 14.75  (0.03) | **0.028** | 0.563 | **1 v 3, 2 v 3,**  **3 v 5** |
| *Erysipelotrichaceae;* Other | 152.43  (0.35) | 64.88  (0.16) | 84.00  (0.19) | 86.63  (0.22) | 104.63  (0.29) | *0.083* | 0.563 | NS |
| *Eubacterium xylanophilum* group | 90.57  (0.23) | 46.75  (0.12) | 106.25  (0.24) | 173.13  (0.43) | 46.25  (0.12) | *0.066* | 0.563 | NS |
| *Lachnospiraceae;*  GCA-900066575 | 153.00  (0.35) | 150.88  (0.38) | 110.63  (0.26) | 109.63  (0.30) | 88.25  (0.25) | 0.463 | 0.707 | NS |
| *Intestinimonas* | 86.29  (0.22) | 64.50  (0.16) | 44.00  (0.11) | 70.38  (0.18) | 64.63  (0.18) | 0.627 | 0.760 | NS |
| *Lachnoclostridium* | 574.93  (1.45) | 443.75  (1.10) | 534.88  (1.24) | 671.38  (1.78) | 570.75  (1.50) | 0.530 | 0.754 | NS |
| *Lachnospiraceae* NK4A136 group | 6,613.21  (17.27) | 5,770.88  (14.21) | 9,396.13  (21.71) | 8,896.13  (22.78) | 7,411.25  (21.20) | **0.009** | 0.563 | **1 v 3, 2 v 3,**  **3 v 5** |
| *Lachnospiraceae* UCG-001 | 5,330.36  (12.90) | 5,490.75  (12.70) | 1,738.88  (4.10) | 1,625.00  (4.22) | 3,238.50  (9.22) | **0.023** | 0.563 | **1 v 4, 2 v 4** |
| *Lachnospiraceae* UCG-006 | 241.07  (0.59) | 194.88  (0.46) | 341.13  (0.78) | 564.88  (1.50) | 256.63  (0.71) | 0.107 | 0.563 | NS |
| *Lachnospiraceae* UCG-008 | 247.29  (0.63) | 191.63  (0.47) | 171.63  (0.39) | 255.25  (0.69) | 232.13  (0.64) | 0.749 | 0.791 | NS |
| *Lachnospiraceae;* A2 | 3,397.71  (8.62) | 2,709.38  (6.62) | 3,449.50  (8.36) | 2,310.25  (7.61) | 3,128.13  (8.45) | 0.481 | 0.708 | NS |
| *Lachnospiraceae;* Other | 3,466.79  (8.61) | 4,435.38  (10.82) | 3,413.13  (8.01) | 3,163.63  (8.69) | 3,364.13  (9.32) | *0.092* | 0.563 | NS |
| *Lactobacillaceae;* Other | 107  (0.25) | 111.75  (0.26) | 140.13  (0.31) | 109.88  (0.27) | 100.38  (0.28) | 0.562 | 0.760 | NS |
| *Lactobacillus* | 4,518.36  (10.42) | 4,532.13  (10.59) | 5,742.25  (12.76) | 4,528.38  (10.83) | 4,086.75  (10.93) | 0.658 | 0.760 | NS |
| *Marvinbryantia* | 391.71  (0.90) | 212.38  (0.52) | 332.75  (0.75) | 344.63  (0.88) | 238.75  (0.66) | 0.217 | 0.563 | NS |
| *Mucispirillum* | 392.21  (1.03) | 546.63  (1.33) | 692.88  (1.73) | 204.75  (0.66) | 412.13  (1.10) | 0.219 | 0.563 | NS |
| *Muribaculaceae;* Other | 5,325.36  (11.78) | 5,082.38  (12.08) | 6,310.25  (13.27) | 5,471.00  (13.32) | 4,757.38  (12.30) | 0.737 | 0.787 | NS |
| *Muribaculum* | 118.43  (0.26) | 57  (0.14) | 84.25  (0.18) | 50.88  (0.12) | 76.5  (0.20) | 0.288 | 0.563 | NS |
| *Negativibacillus* | 55.07  (0.14) | 51.88  (0.14) | 68.88  (0.16) | 73.75  (0.19) | 42.38  (0.10) | 0.101 | 0.563 | NS |
| *Oscillibacter* | 226.93  (0.56) | 230.50  (0.57) | 303.50  (0.71 | 262.13  (0.69) | 294.00  (0.81) | 0.409 | 0.666 | NS |
| *Parabacteroides* | 28.14  (0.06) | 7.00  (0.02) | 25.50  (0.05) | 21.50  (0.05) | 13.63  (0.03) | 0.153 | 0.563 | NS |
| *Parasutterella* | 331.00  (0.74) | 297.75  (0.70) | 453.75  (0.98) | 249.50  (0.59) | 235.88  (0.64) | 0.317 | 0.564 | NS |
| *Rikenellaceae* RC9 gut group | 176.71  (0.38) | 112.25  (0.27) | 160.25  (0.33) | 193.75  (0.47) | 107.00  (0.26) | 0.726 | 0.783 | NS |
| *Rikenellaceae;* Other | 42.93  (0.10) | 38.13  (0.09) | 51.25  (0.12) | 52.88  (0.13) | 40.75  (0.11) | 0.555 | 0.760 | NS |
| *Roseburia* | 1,327.79  (3.39) | 3,945.00  (10.22) | 1,170.75  (2.81) | 1,514.63  (4.36) | 1,328.75  (4.08) | **0.004** | 0.533 | **1 v 2, 2 v 3,**  **2 v 4, 2 v 5** |
| *Ruminiclostridium* | 581.93  (1.40) | 612.50  (1.48) | 441.13  (1.07) | 402.50  (1.07) | 379.63  (1.11) | *0.095* | 0.563 | NS |
| *Ruminiclostridium* 5 | 159.50  (0.40) | 122.63  (0.31) | 188.38  (0.44) | 142.13  (0.37) | 192.63  (0.51) | 0.265 | 0.563 | NS |
| *Ruminiclostridium* 9 | 361.79  (0.89) | 362.00  (0.88) | 335.75  (0.78) | 368.38  (0.96) | 311.63  (0.87) | 0.215 | 0.563 | NS |
| *Ruminococcaceae* UCG-014 | 59.86  (0.13) | 18.50  (0.05) | 84.63  (0.19) | 70.50  (0.18) | 42.25  (0.12) | **0.043** | 0.563 | **2 v 3, 2 v 4** |
| *Ruminococcaceae;* Other | 353.14  (0.87) | 285.25  (0.69) | 329.75  (0.77) | 301.00  (0.77) | 228.25  (0.64) | 0.272 | 0.563 | NS |
| *Ruminococcus* 1 | 61.57  (0.15) | 85.00  (0.20) | 48.38  (0.12) | 37.38  (0.10) | 47.88  (0.14) | 0.131 | 0.563 | NS |

Individual taxa differences between groups were assessed for significance using Kruskal-Wallis test on centered log ratio transformed (CLR) data, with false discovery rate (FDR)-corrected *P*-values reported. Adjusted *P*-values were considered significant at: *FDR *P* ˂ 0.05; trends at **P* ˂ 0.05 are reported. Dunn’s multiple group comparison’s post-hoc tests significance indicated ^#^*P* ˂ 0.05. (1) PBS, phosphate-buffered saline; (2) raw, raw cow’s milk; (3) pasteurized, pasteurized cow’s milk; (4) skimmed, skimmed raw cow’s milk; (5) pasteurized + ALP, pasteurized milk spiked with alkaline phosphatase; count = mean number of sequences in defined group; % RA, percent relative abundance in defined group; NS, non-significant.

**Table S2.** Average total count and percent relative abundance of sequences of individual fecal taxa between the different groups at day 31.

| **Taxonomic Levels** | **PBS ^1^**  **Count**  **(% RA)** | **OVA ^2^**  **Count**  **(% RA)** | **Raw ^3^**  **Count**  **(% RA)** | **Pasteurized ^4^**  **Count**  **(% RA)** | **Skimmed ^5^**  **Count**  **(% RA)** | **Pasteurized + ALP ^6^**  **Count**  **(% RA)** | ***P*-value *** | **FDR  *P-v*alue *** | **Dunn’s**  **post-hoc tests ^#^** |
| --- | --- | --- | --- | --- | --- | --- | --- | --- | --- |
| **Phylum** | | | | | | | | | |
| Actinobacteria | 332.17  (0.98) | 904.25  (2.12) | 386.63  (0.97) | 1,519.63  (3.43) | 416.50  (1.03) | 582.38  (1.38) | 0.131 | 0.467 | NS |
| Bacteroidetes | 14,408.50  (39.39) | 16,665.13  (37.26) | 11,555.50  (28.91) | 14,546.00  (33.04) | 9,715.50  (23.34) | 16,346.00  (38.75) | 0.195 | 0.550 | NS |
| Deferribacteres | 30.83  (0.09) | 62.25  (0.14) | 168.13  (0.42) | 218.38  (0.48) | 226.88  (0.61) | 130.00  (0.33) | **0.032** | 0.207 | **1 v 3, 1 v 5, 2 v 3, 2 v 5** |
| Firmicutes | 19,686.83  (58.60) | 26,410.63  (58.82) | 27,969.25  (69.24) | 26,690.50  (60.90) | 30,058.13  (74.38) | 23,946.25  (57.54) | 0.124 | 0.467 | NS |
| Proteobacteria | 312.17  (0.76) | 575.13  (1.33) | 179.88  (0.44) | 910.38  (2.06) | 244.00  (0.60) | 811.50  (1.93) | **0.000** | **0.008** | **1 v 4, 1 v 6, 2 v 4, 2 v 6, 3 v 4, 3 v 6, 4 v 5, 5 v 6** |
| Tenericutes | 56.17  (0.15) | 134.13  (0.30) | 2.38  (0.01) | 16.50  (0.04) | 11.38  (0.03) | 13.00  (0.03) | **0.005** | *0.074* | **1 v 2, 2 v 3, 2 v 4, 2 v 5, 3 v 6** |
| **Family** | | | | | | | | | |
| Atopobiaceae | 89.33  (0.20) | 242.63  (0.53) | 114.88  (0.29) | 250.50  (0.60) | 202.25  (0.50) | 322.00  (0.76) | 0.294 | 0.599 | NS |
| Bacteroidaceae | 3,905.00  (10.67) | 2,177.25  (5.16) | 1,137.63  (2.89) | 2,356.88  (5.16) | 734.13  (1.74) | 1,511.38  (3.61) | *0.052* | 0.457 | NS |
| Bifidobacteriaceae | 0.17  (0.00) | 438.88  (1.09) | 0.25  (0.00) | 1,008.88  (2.24) | 0.25  (0.00) | 0.25  (0.00) | **0.011** | 0.270 | **1 v 4, 3 v 4, 4 v 5, 4 v 6** |
| Burkholderiaceae | 280.33  (0.68) | 170.63  (0.37) | 55.13  (0.14) | 786.25  (1.78) | 132.13  (0.32) | 657.63  (1.56) | **0.000** | **0.005** | **1 v 4, 2 v 4, 2 v 6, 3 v 4, 3 v 6, 4 v 5, 5 v 6** |
| Clostridiaceae 1 | 346.50  (0.97) | 260.63  (0.58) | 294.00  (0.78) | 199.38  (0.44) | 171.63  (0.40) | 220.38  (0.51) | 0.721 | 0.768 | NS |
| Clostridiales vadin BB60 group | 150.50  (0.43) | 249.13  (0.55) | 119.13  (0.30) | 191.63  (0.44) | 259.75  (0.62) | 198.63  (0.47) | *0.082* | 0.537 | NS |
| Deferribacteraceae | 30.83  (0.09) | 62.25  (0.14) | 168.13  (0.42) | 218.38  (0.48) | 226.88  (0.61) | 130.00  (0.33) | *0.095* | 0.571 | NS |
| Desulfovibrionaceae | 16.00  (0.05) | 78.50  (0.17) | 98.63  (0.24) | 68.63  (0.16) | 93.13  (0.24) | 146.38  (0.35) | **0.010** | 0.264 | **1 v 2, 1 v 3, 1 v 5, 1 v 6, 4 v 6** |
| Eggerthellaceae | 241.50  (0.78) | 220.13  (0.49) | 270.00  (0.67) | 255.88  (0.58) | 213.13  (0.52) | 257.63  (0.61) | 0.375 | 0.599 | NS |
| Enterobacteriaceae | 12.17  (0.03) | 322.50  (0.77) | 24.50  (0.06) | 47.75  (0.11) | 15.75  (0.04) | 2.25  (0.01) | **0.022** | 0.386 | **1 v 6, 1 v 5, 2 v 5, 2 v 6** |
| Erysipelotrichaceae | 246.33  (0.83) | 422.25  (1.00) | 140.13  (0.34) | 412.50  (0.90) | 408.00  (0.98) | 395.25  (0.95) | *0.099* | 0.581 | NS |
| Lachnospiraceae | 6,613.83  (23.71) | 11,207.75  (25.09) | 17,018.25  (41.96) | 13,899.50  (31.75) | 19,457.38  (49.08) | 14,517.38  (34.94) | **0.003** | 0.104 | **1 v 3, 1 v 4, 1 v 5, 1 v 6, 2 v 3, 2 v 5** |
| Lactobacillaceae | 11,391.67  (29.25) | 12,635.00  (27.90) | 8,182.63  (20.49) | 10,046.88  (22.95) | 7,554.00  (17.79) | 6,994.38  (16.78) | 0.271 | 0.599 | NS |
| Muribaculaceae | 8,582.83  (23.75) | 11,690.38  (25.78) | 8,746.63  (21.82) | 10,195.38  (23.37) | 7,294.38  (17.59) | 12,542.38  (29.70) | 0.360 | 0.599 | NS |
| Peptococcaceae | 18.67  (0.07) | 41.38  (0.09) | 42.00  (0.10) | 54.13  (0.12) | 53.13  (0.13) | 45.75  (0.11) | **0.034** | 0.386 | **1 v 4, 1 v 5, 1 v 6** |
| Prevotellaceae | 303.83  (0.78) | 421.25  (0.90) | 355.63  (0.90) | 428.13  (1.03) | 199.75  (0.47) | 676.38  (1.62) | *0.068* | 0.508 | NS |
| Rikenellaceae | 1,500.17  (3.91) | 2,236.75  (5.08) | 1,266.25  (3.18) | 1,450.25  (3.23) | 1,410.38  (3.36) | 1,553.25  (3.68) | 0.401 | 0.599 | NS |
| Ruminococcaceae | 797.33  (3.00) | 1,448.25  (3.26) | 2,073.13  (5.01) | 1,729.00  (3.93) | 2,069.88  (5.18) | 1,490.00  (3.58) | **0.033** | 0.386 | **1 v 3, 1 v 4, 1 v 5** |
| **Genus** | | | | | | | | | |
| *Alistipes* | 1,252.83  (3.33) | 1,862.75  (4.20) | 1,085.38  (2.72) | 1,281.13  (2.86) | 1,250.50  (2.99) | 1,377.63  (3.26) | 0.105 | 0.529 | NS |
| *Alloprevotella* | 286.33  (0.73) | 379.00  (0.81) | 340.88  (0.87) | 409.38  (0.98) | 189.50  (0.45) | 644.13  (1.54) | *0.057* | 0.423 | NS |
| *Anaerotruncus* | 73.83  (0.31) | 96.38  (0.22) | 199.25  (0.49) | 141.63  (0.32) | 213.50  (0.54) | 127.75  (0.31) | *0.092* | 0.500 | NS |
| *ASF356* | 196.83  (0.53) | 294.50  (0.66) | 345.38  (0.85) | 359.25  (0.80) | 383.75  (0.95) | 245.38  (0.59) | 0.491 | 0.632 | NS |
| *Bacteroidaceae;* Other | 106.00  (0.29) | 58.75  (0.14) | 30.13  (0.08) | 56.50  (0.12) | 21.13  (0.05) | 39.38  (0.09) | **0.014** | 0.163 | **1 v 3, 1 v 5, 2 v 5** |
| *Bacteroides* | 3799.00  (10.38) | 2,118.50  (5.02) | 1,107.50  (2.81) | 2,300.38  (5.03) | 713.00  (1.69) | 1472.00  (3.51) | **0.016** | 0.171 | **1 v 3, 1 v 5, 2 v 5** |
| *Bifidobacterium* | 0.17  (0.00) | 419.00  (1.04) | 0.13  (0.00) | 966.00  (2.15) | 0.25  (0.00) | 0.13  (0.00) | **0.014** | 0.162 | **1 v 4, 3 v 4, 4 v 5, 4 v 6** |
| *Bilophila* | 15.83  (0.05) | 35.38  (0.08) | 97.25  (0.23) | 68.13  (0.15) | 92.63  (0.24) | 43.25  (0.10) | **0.008** | 0.118 | **1 v 3, 1 v 5, 2 v 3, 2 v 5** |
| *Blautia* | 22.00  (0.08) | 160.38  (0.37) | 216.00  (0.54) | 388.88  (0.87) | 99.25  (0.23) | 121.13  (0.29) | **0.003** | *0.066* | **1 v 2, 1 v 3 1 v 4, 1 v 6, 3 v 5** |
| *Butyricicoccus* | 23.00  (0.09) | 43.38  (0.10) | 102.13  (0.25) | 52.13  (0.12) | 69.13  (0.18) | 84.75  (0.20) | **0.008** | 0.117 | **1 v 3, 1 v 6, 2 v 3, 2 v 6** |
| *Candidatus Arthromitus* | 332.67  (0.93) | 251.88  (0.56) | 285.13  (0.75) | 192.75  (0.43) | 165.13  (0.38) | 210.63  (0.49) | 0.328 | 0.590 | NS |
| *Candidatus Stoquefichus* | 2.33  (0.01) | 63.13  (0.14) | 3.88  (0.01) | 79.88  (0.17) | 88.13  (0.21) | 144.25  (0.34) | **0.006** | *0.097* | **1 v 2, 1 v 4, 1 v 5, 1 v 6, 2 v 3, 3 v 4, 3 v 5, 3 v 6** |
| *Clostridiales vadin* BB60 group; Other | 150.50  (0.43) | 249.13  (0.55) | 119.13  (0.30) | 191.63  (0.44) | 259.75  (0.62) | 198.63  (0.47) | *0.052* | 0.397 | NS |
| *Coriobacteriaceae* UCG-002 | 87.00  (0.19) | 236.50  (0.52) | 110.25  (0.28) | 243.75  (0.58) | 197.63  (0.49) | 314.38  (0.74) | 0.311 | 0.590 | NS |
| *Enterorhabdus* | 186.50  (0.62) | 186.75  (0.42) | 200.13  (0.50) | 213.63  (0.48) | 179.00  (0.44) | 220.00  (0.52) | 0.392 | 0.590 | NS |
| *Erysipelatoclostridium* | 60.50  (0.29) | 130.50  (0.33) | 22.75  (0.06) | 116.88  (0.25) | 87.50  (0.20) | 80.00  (0.19) | 0.334 | 0.590 | NS |
| *Erysipelotrichaceae;*  Other | 183.00  (0.53) | 168.00  (0.38) | 113.38  (0.28) | 103.75  (0.23) | 231.75  (0.56) | 170.75  (0.42) | *0.072* | 0.462 | NS |
| *Eubacterium xylanophilum* group | 41.33  (0.14) | 32.63  (0.07) | 106.75  (0.26) | 55.63  (0.13) | 28.38  (0.07) | 63.88  (0.15) | 0.347 | 0.590 | NS |
| *Lachnospiraceae;*  GCA-900066575 | 57.83  (0.25) | 90.75  (0.20) | 102.25  (0.25) | 94.63  (0.21) | 79.38  (0.20) | 53.13  (0.13) | 0.135 | 0.590 | NS |
| *Intestinimonas* | 25.17  (0.09) | 43.13  (0.10) | 55.88  (0.14) | 46.50  (0.10) | 91.13  (0.23) | 39.88  (0.10) | **0.027** | 0.239 | **1 v 5, 2 v 5, 4 v 5, 5 v 6** |
| *Lachnoclostridium* | 285.50  (0.98) | 372.13  (0.83) | 515.75  (1.27) | 505.25  (1.14) | 497.25  (1.22) | 456.75  (1.10) | 0.549 | 0.645 | NS |
| *Lachnospiraceae* NK4A136 group | 1,608.33  (5.87) | 4,399.75  (9.77) | 6,321.13  (15.59) | 6,045.00  (13.78) | 9,674.00  (24.60) | 5,868.88  (14.00) | **0.001** | **0.038** | **1 v 3, 1 v 4, 1 v 5, 1 v 6, 2 v 5** |
| *Lachnospiraceae* UCG-001 | 1,175.67  (4.40) | 796.13  (1.80) | 2,478.63  (6.10) | 787.38  (1.79) | 2,000.75  (5.06) | 2,200.00  (5.40) | **0.003** | *0.066* | **2 v 3, 2 v 5, 2 v 6, 3 v 4, 4 v 5, 4 v 6** |
| *Lachnospiraceae* UCG-006 | 107.67  (0.34) | 200.38  (0.44) | 300.13  (0.73) | 258.13  (0.60) | 279.00  (0.69) | 243.88  (0.58) | 0.300 | 0.590 | NS |
| *Lachnospiraceae* UCG-008 | 31.50  (0.13) | 95.38  (0.21) | 162.38  (0.40) | 110.25  (0.25) | 172.75  (0.42) | 157.75  (0.38) | **0.002** | *0.054* | **1 v 2, 1 v 3, 1 v 4, 1 v 5, 1 v 6, 2 v 3** |
| *Lachnospiraceae;* A2 | 286.33  (0.90) | 754.13  (1.76) | 621.00  (1.55) | 608.63  (1.38) | 770.88  (1.97) | 721.75  (1.74) | 0.281 | 0.590 | NS |
| *Lachnospiraceae;*  Other | 1,106.50  (4.01) | 1,998.00  (4.45) | 2,681.63  (6.64) | 2,311.63  (5.31) | 3,162.38  (7.85) | 2,419.38  (5.84) | **0.019** | 0.185 | **1 v 3, 1 v 4, 1 v 5, 1 v 6** |
| *Lactobacillaceae;*  Other | 266.00  (0.69) | 292.25  (0.64) | 181.63  (0.46) | 232.00  (0.53) | 176.63  (0.42) | 164.50  (0.39) | *0.076* | 0.467 | NS |
| *Lactobacillus* | 11,125.50  (28.57) | 12,342.38  (27.25) | 8,000.50  (20.03) | 9,814.38  (22.42) | 7,377.00  (17.37) | 6,829.50  (16.38) | *0.089* | 0.493 | NS |
| *Marvinbryantia* | 526.33  (1.47) | 532.50  (1.20) | 428.25  (1.07) | 394.50  (0.88) | 450.88  (1.08) | 474.25  (1.12) | 0.389 | 0.590 | NS |
| *Mucispirillum* | 29.67  (0.09) | 60.25  (0.14) | 160.88  (0.40) | 212.00  (0.47) | 219.88  (0.59) | 124.88  (0.31) | 0.236 | 0.590 | NS |
| *Muribaculaceae;*  Other | 8,265.50  (22.91) | 11,504.63  (25.37) | 8,569.13  (21.38) | 9,993.38  (22.91) | 7,156.13  (17.26) | 12,246.88  (29.00) | 0.146 | 0.590 | NS |
| *Muribaculum* | 317.33  (0.84) | 185.75  (0.41) | 177.50  (0.45) | 202.00  (0.47) | 138.25  (0.33) | 295.50  (0.70) | 0.195 | 0.590 | NS |
| *Negativibacillus* | 35.50  (0.10) | 53.88  (0.13) | 44.63  (0.11) | 46.63  (0.11) | 29.00  (0.07) | 28.25  (0.07) | 0.475 | 0.616 | NS |
| *Oscillibacter* | 42.00  (0.15) | 144.88  (0.32) | 179.63  (0.43) | 220.63  (0.50) | 308.50  (0.78) | 130.63  (0.32) | **0.003** | *0.070* | **1 v 2, 1 v 3, 1 v 4, 1 v 5, 5 v 6** |
| *Parabacteroides* | 102.33  (0.24) | 116.50  (0.29) | 38.50  (0.10) | 97.88  (0.22) | 64.75  (0.15) | 49.13  (0.12) | 0.352 | 0.590 | NS |
| *Parasutterella* | 273.67  (0.66) | 165.63  (0.36) | 54.00  (0.14) | 766.00  (1.73) | 127.75  (0.31) | 637.88  (1.51) | **0.000** | **0.004** | **1 v 3, 2 v 4, 2 v 6, 3 v 4, 3 v 6, 4 v 5, 5 v 6** |
| *Rikenellaceae* RC9 gut group | 203.83  (0.47) | 314.88  (0.75) | 145.25  (0.37) | 129.38  (0.28) | 118.13  (0.27) | 130.25  (0.31) | *0.073* | 0.462 | NS |
| *Rikenellaceae;* Other | 43.33  (0.10) | 59.13  (0.14) | 35.50  (0.09) | 39.75  (0.09) | 41.75  (0.10) | 45.38  (0.11) | 0.164 | 0.590 | NS |
| *Roseburia* | 1,124.33  (4.43) | 1,384.38  (3.10) | 2,646.00  (6.48) | 1,906.50  (4.43) | 1,740.75  (4.44) | 1,406.63  (3.41) | 0.191 | 0.590 | NS |
| *Ruminiclostridium* | 169.33  (0.73) | 267.50  (0.59) | 638.00  (1.53) | 394.63  (0.90) | 345.50  (0.87) | 337.88  (0.81) | 0.142 | 0.590 | NS |
| *Ruminiclostridium* 5 | 34.00  (0.13) | 51.13  (0.12) | 116.88  (0.28) | 96.88  (0.22) | 164.25  (0.41) | 83.38  (0.20) | **0.002** | *0.054* | **1 v 3, 1 v 4, 1 v 5, 2 v 3, 2 v 4, 2 v 5** |
| *Ruminiclostridium* 9 | 162.67  (0.59) | 268.88  (0.60) | 323.00  (0.78) | 307.50  (0.70) | 397.25  (0.99) | 273.50  (0.66) | **0.042** | 0.342 | **1 v 3, 1 v 5, 2 v 5, 5 v 6** |
| *Ruminococcaceae* UCG-014 | 70.50  (0.19) | 69.50  (0.16) | 43.63  (0.10) | 58.63  (0.13) | 34.25  (0.08) | 37.50  (0.09) | *0.075* | 0.467 | NS |
| *Ruminococcaceae;*  Other | 124.33  (0.48) | 238.88  (0.54) | 277.88  (0.67) | 273.88  (0.62) | 316.63  (0.79) | 247.50  (0.59) | 0.105 | 0.529 | NS |
| *Ruminococcus* 1 | 17.17  (0.06) | 23.88  (0.05) | 37.25  (0.09) | 28.38  (0.06) | 39.75  (0.10) | 24.50  (0.06) | *0.097* | 0.520 | NS |

Individual taxa differences between groups were assessed for significance using Kruskal-Wallis test on centered log ratio transformed (CLR) data, with false discovery rate (FDR)-corrected *P*-values reported. Adjusted *P*-values were considered significant at *FDR *P* ˂ 0.05; trends at: **P* ˂ 0.05 are reported. Dunn’s multiple group comparison’s post-hoc tests significance indicated ^#^*P* ˂ 0.05. (1) PBS, phosphate-buffered saline; (2) OVA, ovalbumin; (3) raw, raw cow’s milk; (4) pasteurized, pasteurized cow’s milk; (5) skimmed, skimmed raw cow’s milk; (6) pasteurized + ALP, pasteurized milk spiked with alkaline phosphatase; count = mean number of sequences in defined group; % RA, percent relative abundance in defined group; NS, non-significant.
