# Supplementary figures and images for: Raw Milk-Induced Protection against Food Allergic Symptoms in Mice Is Accompanied by Shifts in Microbial Community Structure
Source: Int J Mol Sci. 2021 Mar 26;22(7):3417. doi: 10.3390/ijms22073417 (PMC8037148; doi:10.3390/ijms22073417)

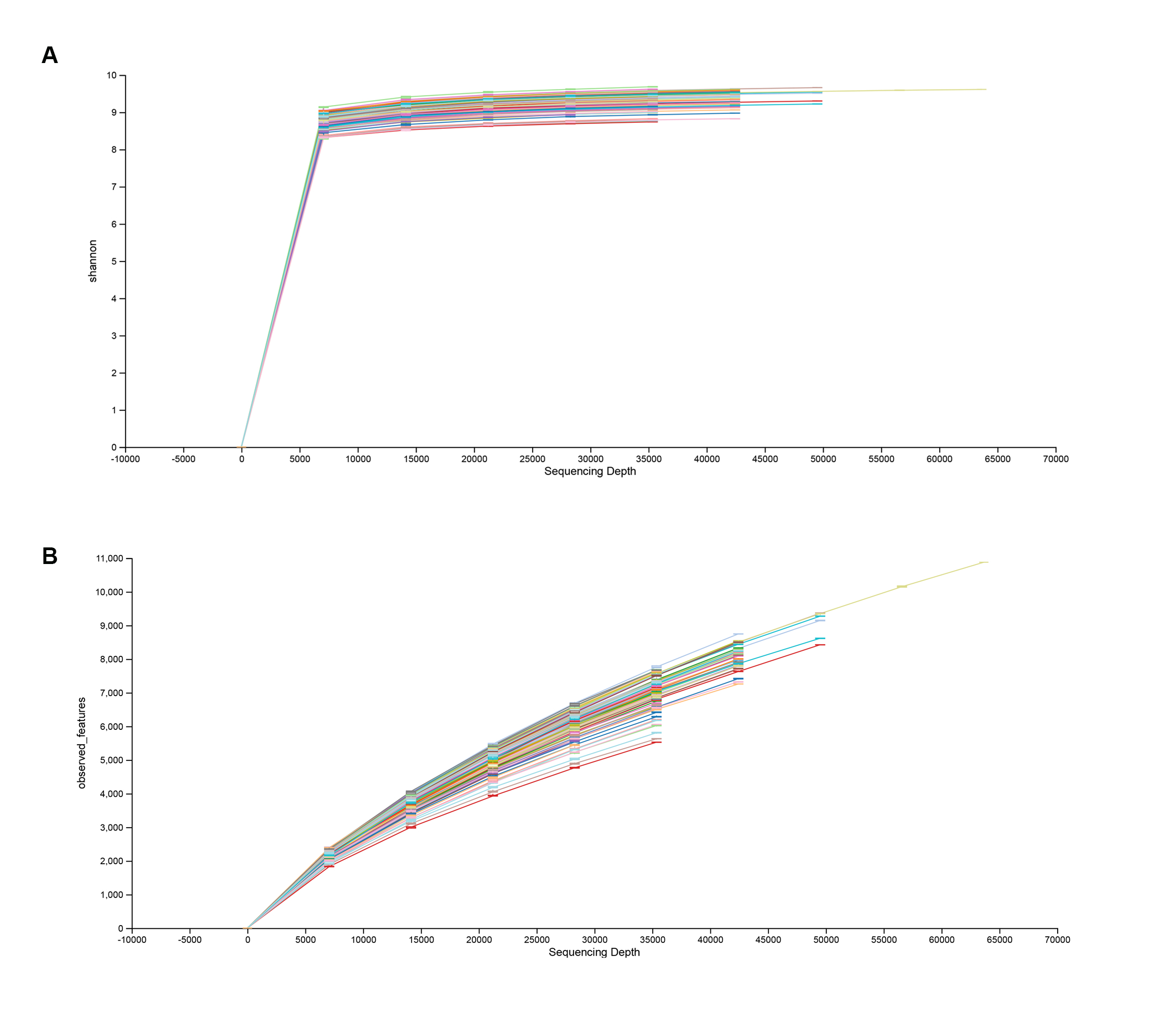

Supplement: Supplementary file 1 [file ijms-22-03417-s001.zip › Figure S1.tif]

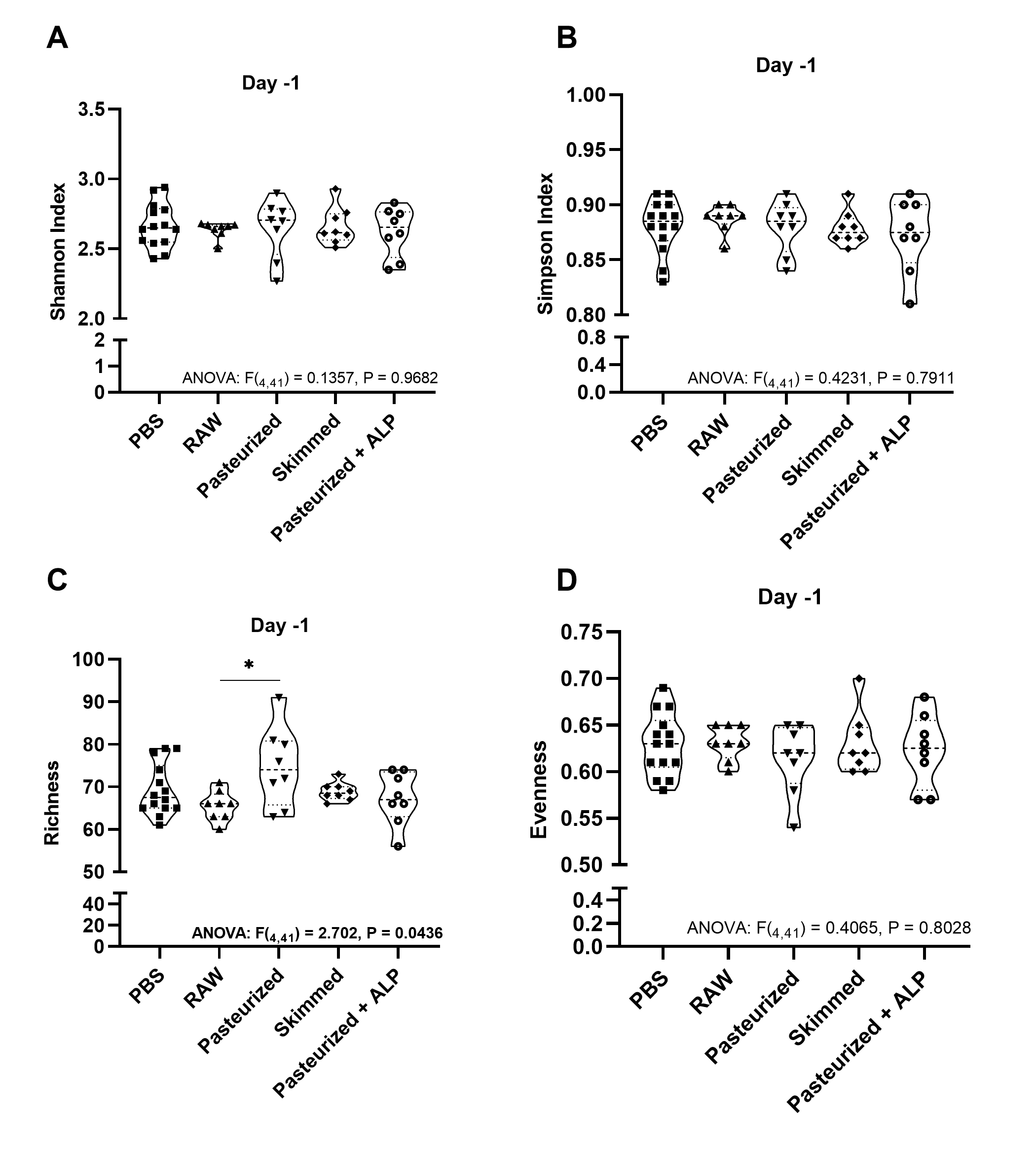

Supplement: Supplementary file 1 [file ijms-22-03417-s001.zip › Figure S2.tif]

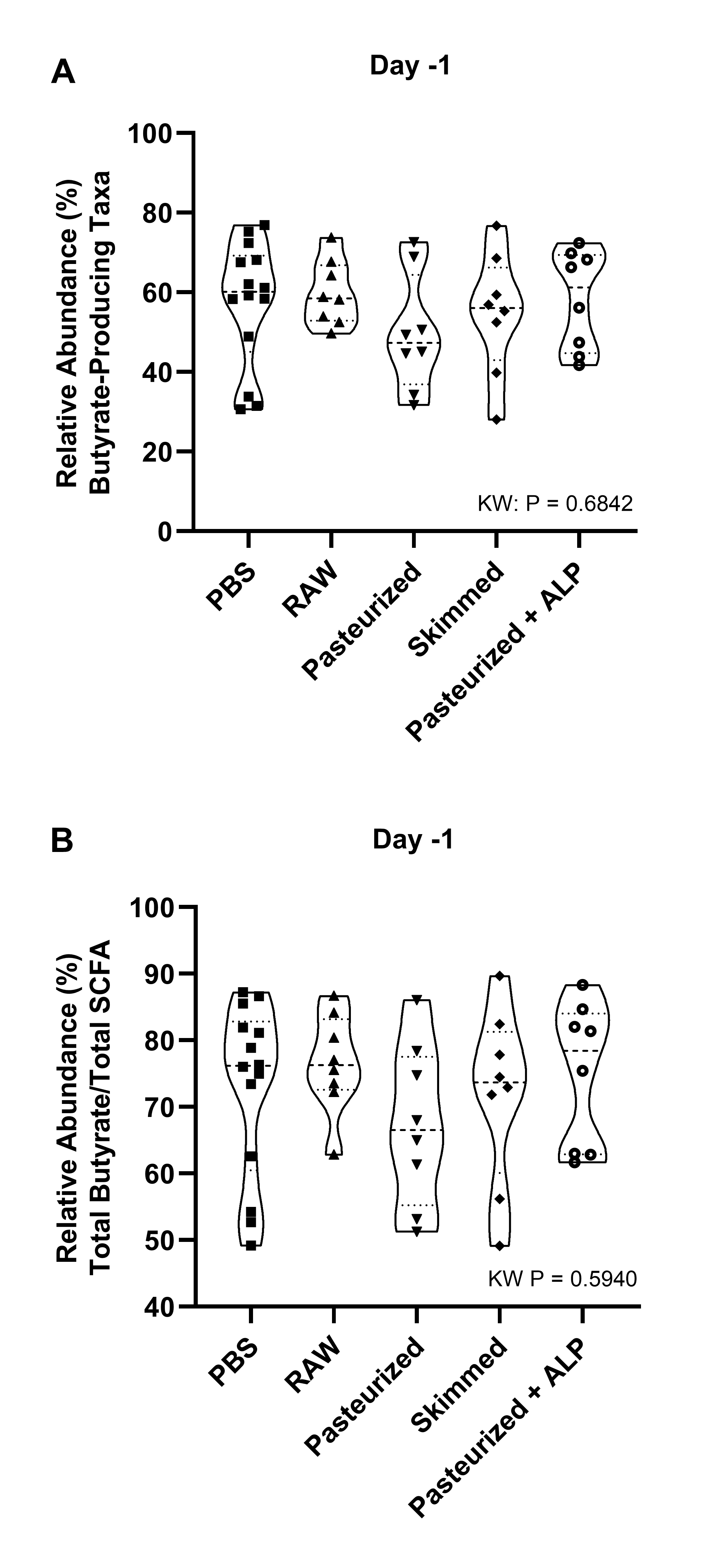

Supplement: Supplementary file 1 [file ijms-22-03417-s001.zip › Figure S3.tif]

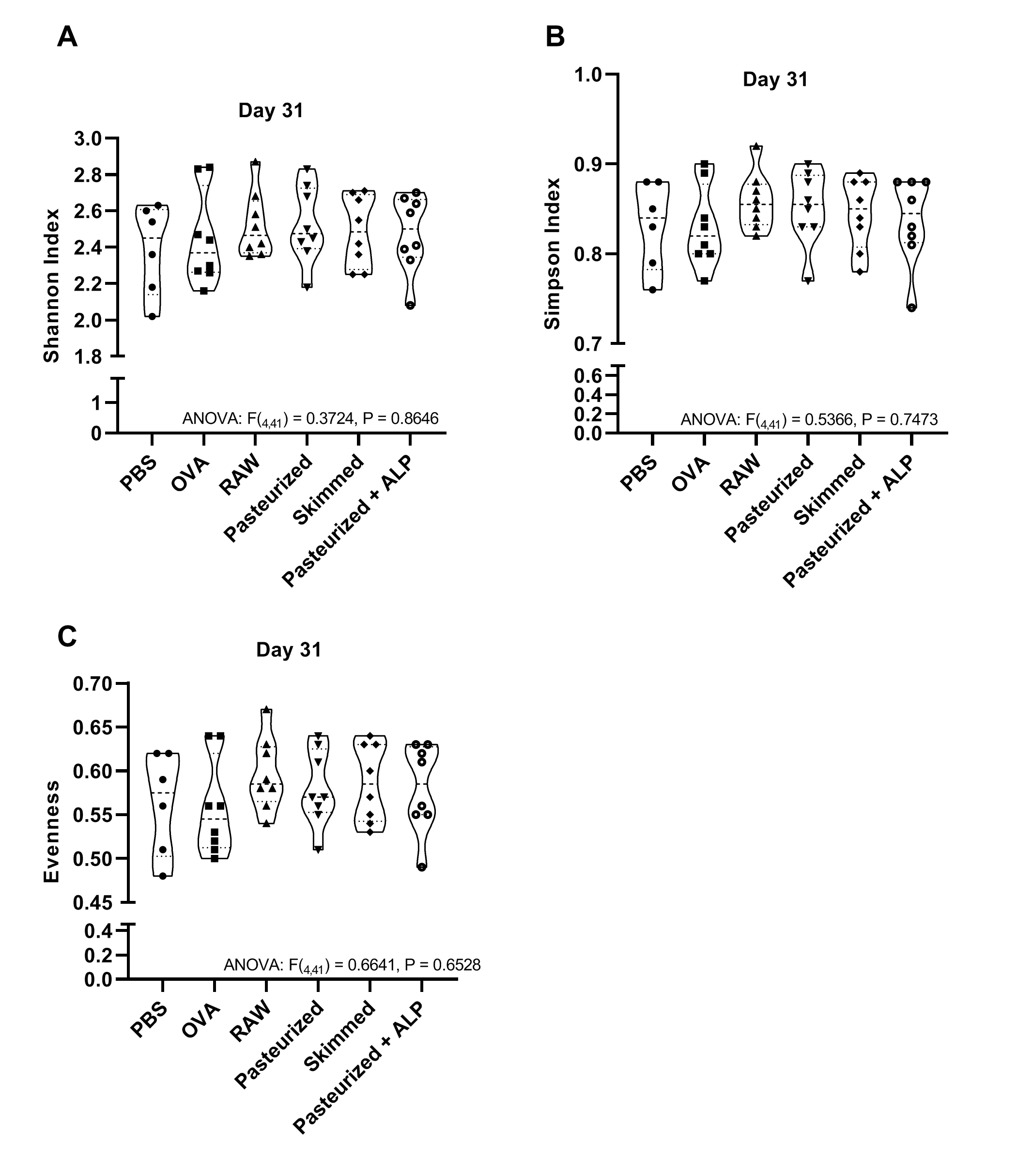

Supplement: Supplementary file 1 [file ijms-22-03417-s001.zip › Figure S4.tif]

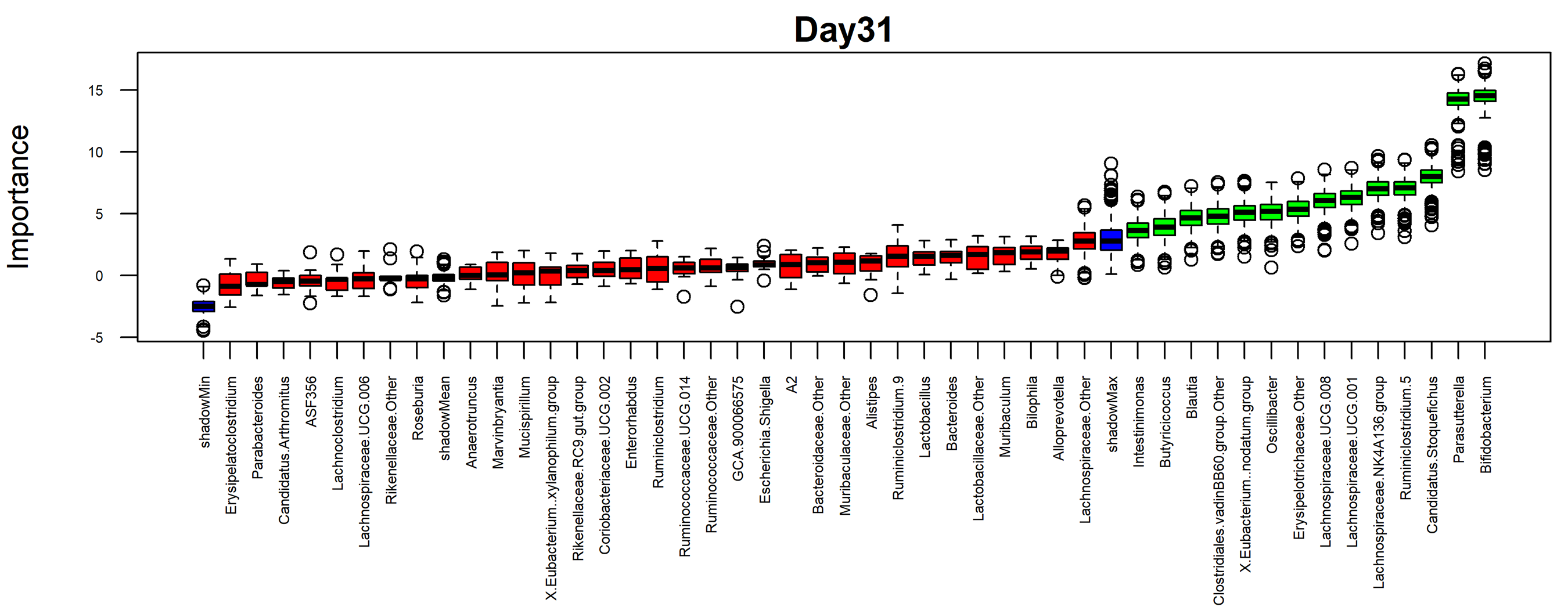

Supplement: Supplementary file 1 [file ijms-22-03417-s001.zip › Figure S5.tif]
